# Tips and Tricks for Installation of the SLIM Nail in Osteogenesis Imperfecta with Narrow Medullary Canals: A Surgical Guide with Case Insights

**DOI:** 10.3390/children12091190

**Published:** 2025-09-07

**Authors:** Peter Joseph Mounsef, Jack Legler, Reggie Hamdy

**Affiliations:** 1 Shriners Hospitals for Children, Montreal, QC H4A 0A9, Canada; 2 Faculty of Medicine & Health Sciences, McGill University, Montreal, QC H3A 0G4, Canada; 3 Division of Pediatric Orthopaedic Surgery, Montreal Children’s Hospital, Montreal, QC H4A 3H9, Canada

**Keywords:** SLIM nail, osteogenesis imperfecta, intramedullary rodding, deformity correction

## Abstract

**Highlights:**

**What are the main findings?**

- The SLIM nail is a viable intramedullary fixation option for children with osteogenesis imperfecta who have narrow bone canals or are nearing skeletal maturity.
- Detailed surgical tips and case-based examples demonstrate strategies to overcome common intraoperative challenges.

**What is the implication of the main finding?**

- Surgeons can use the SLIM nail as an effective alternative when standard telescoping rods are unsuitable.
- Sharing technical insights may improve surgical outcomes and expand treatment options for children with complex bone fragility.

**Abstract:**

Introduction: Osteogenesis imperfecta (OI) presents significant surgical challenges due to bone fragility, narrow medullary canals, and complex deformities. While telescoping rods like the Fassier–Duval (FD) system are commonly used in growing patients, they are unsuitable when the canal diameter is too small or when patients approach skeletal maturity. The Simple Locking Intramedullary (SLIM) nail offers a solid, non-telescoping alternative in these cases. Methods: We describe the surgical technique for SLIM nail implantation and highlight key technical pearls developed through institutional experience, focusing on preoperative planning, intraoperative strategies, and the management of unique anatomical challenges in OI patients. Results: Three cases illustrate the application of these techniques: the first case demonstrates SLIM nail insertion in a 3-year-old child with a narrow IM canal to correct significant bowing; reaming was performed retrograde from the osteotomy site for the proximal segment and antegrade for the distal segment. The second case is a 15-year-old OI patient with a disengaged FD rod and narrow IM canal showing insertion of SLIM rod, and the third case is a 16-year-old patient with femoral deformity and telescoping rod who needed revision with SLIM nail and supplemental plate fixation. Conclusions: The SLIM nail is a viable option for select OI patients.

## 1. Introduction

Osteogenesis imperfecta (OI) is a rare genetic disorder characterized by increased bone fragility, frequent fractures, and progressive limb deformities [1]. This condition is typically caused by mutations in the COL1A1 or COL1A2 genes, which code for the alpha chains of type 1 collagen. Sillence et al. (1979) devised a 4-type classification system for OI based on severity and phenotypic presentation, with type I being mild and not causing bone deformity, type II resulting in fractures in utero and usually death shortly after, type III resulting in fractures at birth and severe physical disabilities, and type IV resulting in moderate bone deformity [2]. The diagnosis of OI is based on clinical and radiological features. Pertinent clinical features include fractures from mild trauma, growth deficiency, and bowing deformities of long bones. Skeletal radiographs reveal osteopenia, long-bone bowing, undertubulation, metaphyseal flaring, and vertebral compressions.

Surgical management in pediatric OI patients aims to correct deformities and prevent fractures, restoring function and improving quality of life. Among surgical options, intramedullary rodding remains the standard surgical intervention of choice for recurrent fractures and severe deformities in children and adolescents with OI, as it protects the whole length of the bone [3,4]. Two types of intra-medullary nails can be used in cases of OI: non-telescoping nails, which are non-lengthening (such as Kirshner wires, Rush rods, Elastic nails, and SLIM nails), and telescoping or lengthening rods. The telescoping rods have been shown to present several advantages over the non-telescoping rods, specifically regarding the need for revision.

**Figure 1:**
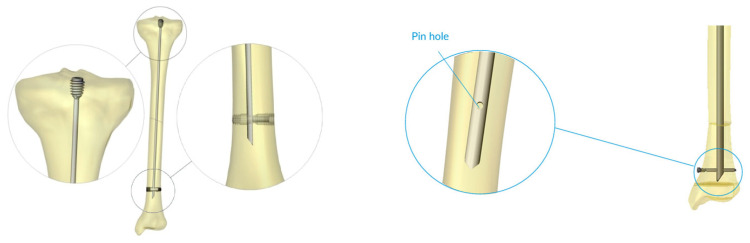
The SLIM nail—a closer look at the proximal threads, distal bullet, and distal pin hole are shown.

**Figure 2:**
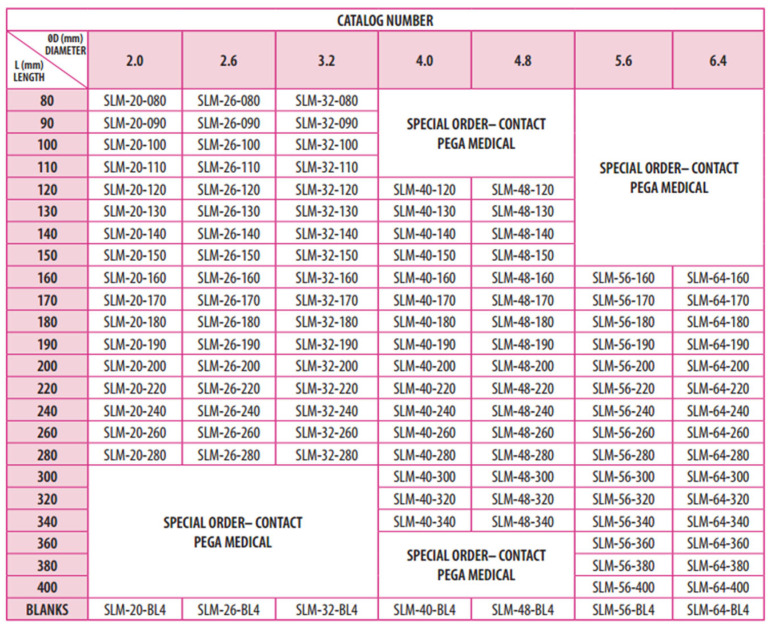
SLIM selection guide from the Pega Medical SLIM nail surgical technique manual.

The Fassier–Duval (FD) rod is one type of telescoping rod that was specifically developed to address the challenges of the growing skeleton, offering a telescoping design that lengthens with the child’s growth and minimizes the need for repeated surgeries [5]. Typically, telescoping rods like the FD are used to maintain long-term alignment and stability. However, the smallest rod diameter is 3.2 mm, which is not suitable for smaller bone canal diameters. In such cases, surgeons have historically used non-telescoping rods such as K-wires or Rush rods, though these are prone to migration and loss of fixation. To overcome this problem, the Simple Locking Intramedullary (SLIM) nail was developed [6]. The SLIM^TM^ nail is manufactured by OrthoPediatrics Canada and was first produced in 2015. It is cleared for use via FDA 510(k) (K143355) [7]. The SLIM nail is a solid, non-telescoping intramedullary nail designed specifically for fixation in long bones with small medullary canals that cannot accommodate larger solid or telescoping nails. The SLIM nail has proximal and distal locking options to help control the stability of the fixation (Figure 1). However, its main advantage over other non-telescoping nails is the presence of threads in the proximal part of the rod (as shown in Figure 1) that provides a better purchase and anchoring in the epiphysis and therefore renders it less liable to migration. It also has a large range of diameters (2.0–6.4 mm) and lengths (80–400 mm) (Figure 2). It can be used in the femur, tibia, humerus, ulna, and fibula in both skeletally immature and mature patients. Despite these benefits, its main disadvantage is its non-telescoping nature, which may allow distal deformity and fracture over time as the child grows, thus indicating the need for revision surgeries.

In this article, we describe the SLIM nail surgical technique, highlight key surgical pearls, and present three illustrative cases from our institutional experience. Our goal is to share practical technical strategies and insights that can assist surgeons in managing the complex anatomical and mechanical challenges encountered when using the SLIM nail in pediatric OI patients with narrow intramedullary canals.

## 2. Methods

### 2.1. Preoperative Planning

**Figure 3:**
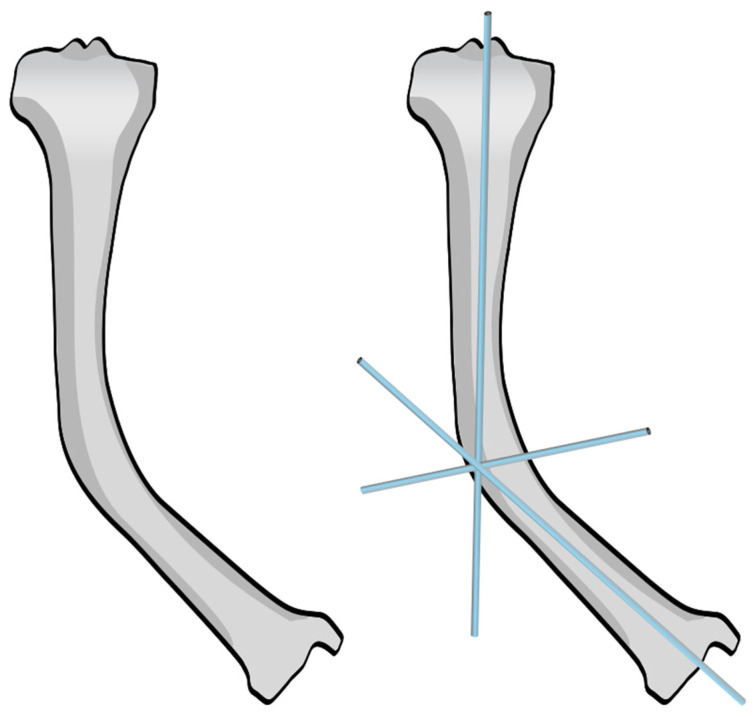
Identification of the CORA in a tibia on an AP view in the coronal plane.

**Figure 4:**
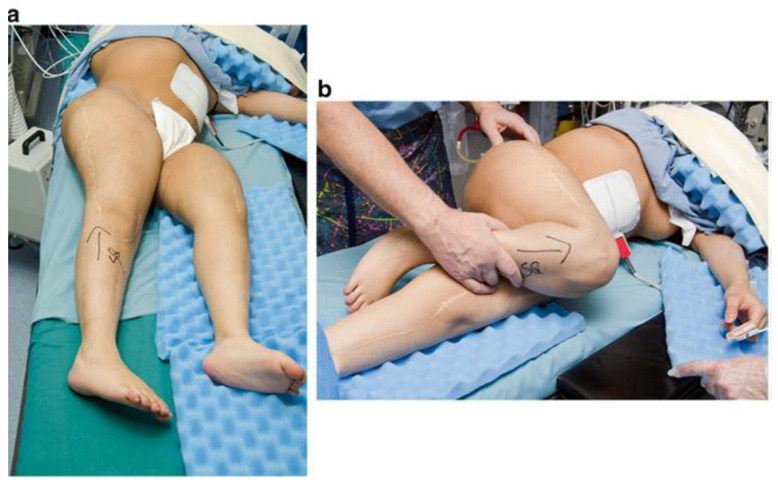
Patient positioned in a supine position with the patella facing forwards (**a**) and with internal rotation of the right hip (**b**).

As for the installation of any intramedullary nails used in cases of OI, careful preoperative planning is crucial. This includes assessment of the size of the canal, length of the bone, degree and direction of deformity, and the number of centers of rotation of angulation (CORAs) (Figure 3). Before beginning the procedure, the patient is placed in a supine position with a cushion placed under the ipsilateral buttock, allowing the limb to rest in internal rotation (Figure 4). This setup provides a clear lateral view of the femur without requiring intraoperative repositioning of the C-arm.

### 2.2. Identification of the Entry Point

**Figure 5:**
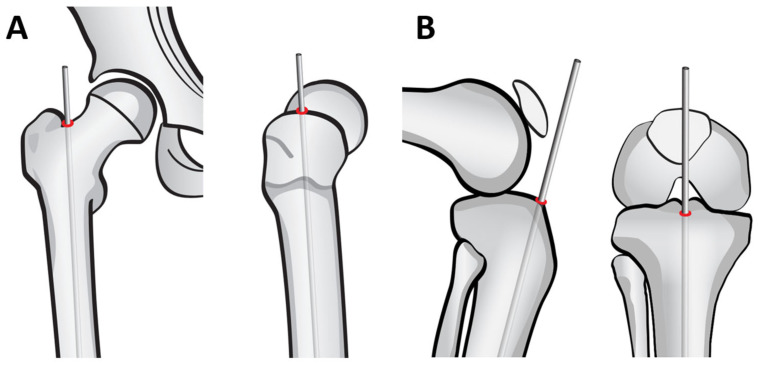
Identification of entry points. (**A**): Femoral entry point. (**B**): Tibial entry point.

Before starting the surgery, it is crucial to identify the rods entry point properly. For the femur, the entry point is located in the piriformis fossa, just adjacent and medial to the border of the greater trochanter. (Figure 5A) The entry point should be in line with the long axis of the femur to avoid malalignment and allow for correct nail positioning. For the tibia, the entry point is usually anterior and in the middle of the tibia. (Figure 5B)

### 2.3. Osteotomy

**Figure 6:**
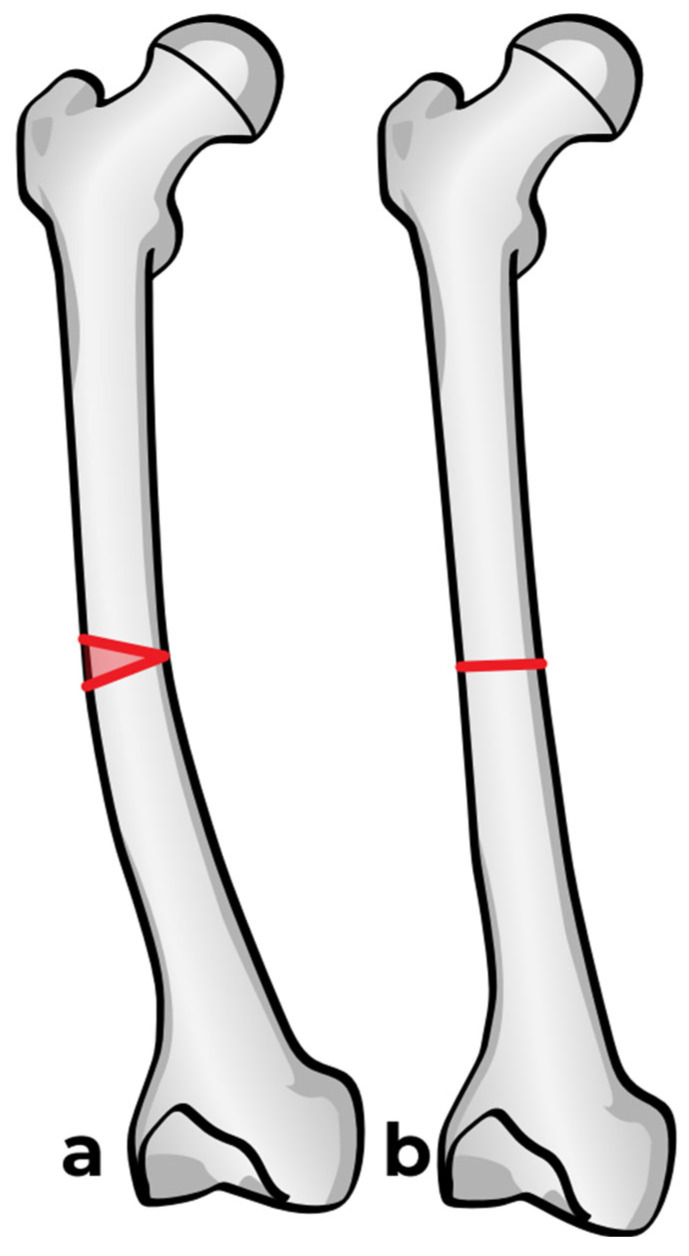

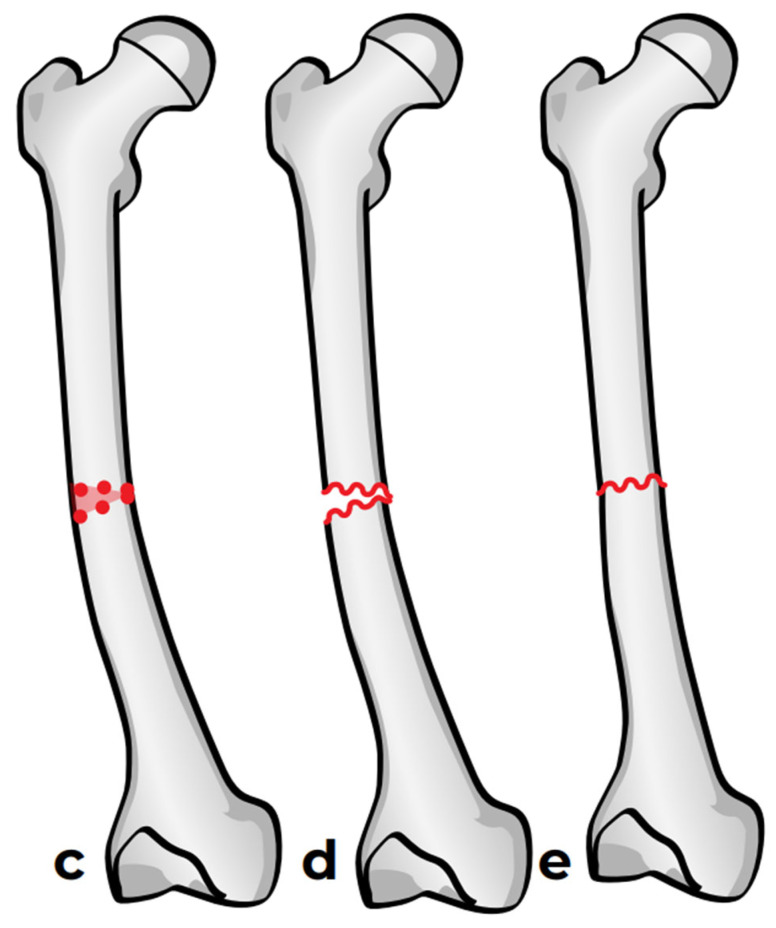
Osteotomy techniques. (**a**,**b**): Osteotomy using an oscillating saw. (**c**–**e**): Osteotomy using multiple drill holes and an osteotome.

Two options are available: the first one is a straight-cut osteotomy using an oscillating saw, which creates a smooth planar surface on either side (Figure 6a). This method, however, provides minimal rotational stability once the bone segments are reduced (Figure 6b). Furthermore, the thermal injury generated by the power saw may have a negative effect on bone healing at the osteotomy site. To address these limitations, we used a multiple drill hole osteotomy technique, in which closely spaced drill holes are made along the planned osteotomy plane before making the cut (Figure 6c). This results in opposing surfaces being rugged and not smooth (Figure 6d). This interdigitation acts as a mechanical interlock between the bone fragments once reduced, which offers some rotational stability and reduces the need for fixation hardware (Figure 6e).

### 2.4. Reaming and Nail Insertion

**Figure 7:**
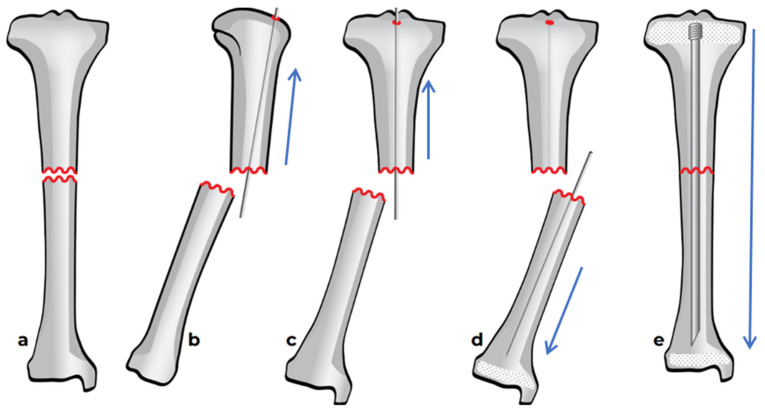
Intramedullary reaming and SLIM nail insertion. Blue arrows indicate the direction of reaming and nail insertion. (**a**) Osteotomy is complete. (**b**) Lateral view of proximal reaming via the osteotomy site. (**c**) AP view of proximal reaming via the osteotomy site. (**d**) AP view of distal reaming via the osteotomy site. (**e**) Insertion of the SLIM nail.

Once the osteotomy is complete, the two bone segments can be reduced to ensure proper alignment (Figure 7a). Reaming can then be performed retrograde into the proximal bone segment through the osteotomy site, and antegrade into the distal bone segment (Figure 7b–d). Under fluoroscopy, external K-wires or SLIM nails are used to measure the required nail length, which is subsequently verified with the actual nail before insertion. Finally, the nail is inserted, positioned centrally in the canal, and advanced until the proximal threads anchor in the epiphysis (Figure 7e). The technique described above and in Figure 7 describes the retrograde approach. In cases with mild deformity (less than 20 degrees in the coronal or sagittal plane) a mini invasive percutaneous osteotomy could be performed, and in such cases, an antegrade approach could be used, in which the insertion of the guide wire, reaming, and insertion of the SLIM nails are all performed through the proximal entry points.

### 2.5. Surgical Pearls

In addition to the core procedural steps, several technical considerations can significantly impact the success and safety of SLIM nail installation in patients with osteogenesis imperfecta (OI). These surgical pearls summarize key insights and recommendations drawn from clinical experience to optimize outcomes and avoid complications.

#### 2.5.1. General Principles

**Load-sharing principle**: The SLIM nail—similar to other intramedullary nails in cases of OI-operates on a load-sharing principle, not a load-bearing principle, making it especially advantageous in osteopenic bone conditions such as OI. By distributing mechanical stress across the bone, it encourages natural bone stimulation and remodeling.

**Avoid oversized nails**: The primary goal of the nail is to protect and align the bone during growth, not to fill or rigidly fix the canal. Oversized, tightly fitted nails can lead to bone resorption, especially in metabolic bone diseases like OI. Selecting the correct diameter is essential.

Preoperative Preparation and Positioning

**Gentle patient handling**: Patients with OI are highly susceptible to fractures. It is critical to handle them gently during transport, positioning, and draping to avoid iatrogenic injuries before surgery even begins.

**Collaborate with implant representatives**: Having a company representative present in the operating room can be invaluable. They can assist with implant selection, guide the surgical team on available instrumentation, and help ensure smooth workflow with unfamiliar devices.

#### 2.5.2. Osteotomy Techniques

**Osteotomy planning with multiple CORAs**: When two deformity points (CORAs) are present, performing a single, well-planned osteotomy between them—if feasible—can correct both deformities simultaneously, simplifying the surgical process and reducing operative time.

**Percutaneous osteotomies**: Percutaneous osteotomies should be carried out using multiple drill holes along the planned osteotomy line, completed with a sharp osteotome. This approach minimizes soft tissue damage and avoids heat-related bone injury.

**Handling minimal vs. large angular deformities**: For minor angular deformities (<20–30°), percutaneous osteotomy without bony resection is typically sufficient. In larger deformities, a formal open osteotomy with wedge resection at the apex is recommended for accurate correction

Technical Considerations During Nail Insertion

**Avoid eccentric reaming**: Eccentric reaming risks breaching the bone cortex and weakening the construct. To prevent this, ensure the bone is correctly realigned and maintain central guidewire placement during reaming, using fluoroscopy when needed.

**Handling sclerotic bone**: In cases of severe bone sclerosis where the medullary canal is narrowed or obliterated, guide pin insertion may be impossible. Small conventional drill bits can be used carefully to create or widen the canal before nail insertion.

**Retrograde vs. Antegrade Insertion**: In the femur and humerus, rods can be inserted retrograde or antegrade. In obese patients, the retrograde approach often provides better access and visualization, especially when proximal landmarks are difficult to palpate. The choice depends on fracture location, anatomy, and surgical goals.

#### 2.5.3. Additional Stabilization

**Managing rotational instability**: In cases of rotational instability, applying a unicortically placed mini-fragment plate (2.7 mm) at the osteotomy site can improve rotational stability and promote bone healing. Plan to remove the plate after healing is complete to prevent stress risers that could predispose the bone to future fractures.

## 3. Results—Case Series

**Figure 8:**
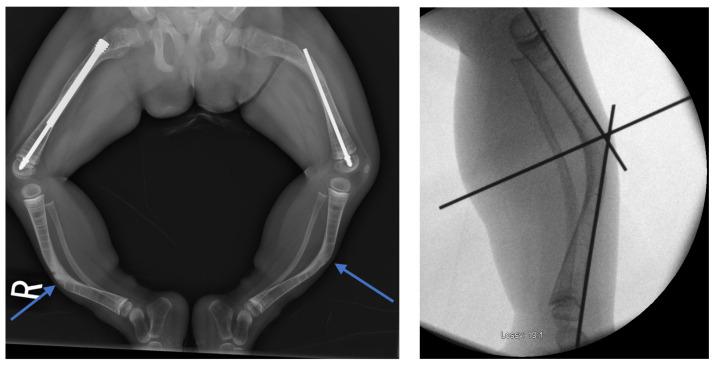
Preoperative and intraoperative X-rays of the lower extremities showing tibia deformities/fractures. Blue arrows indicate the CORAs.

**Figure 9:**
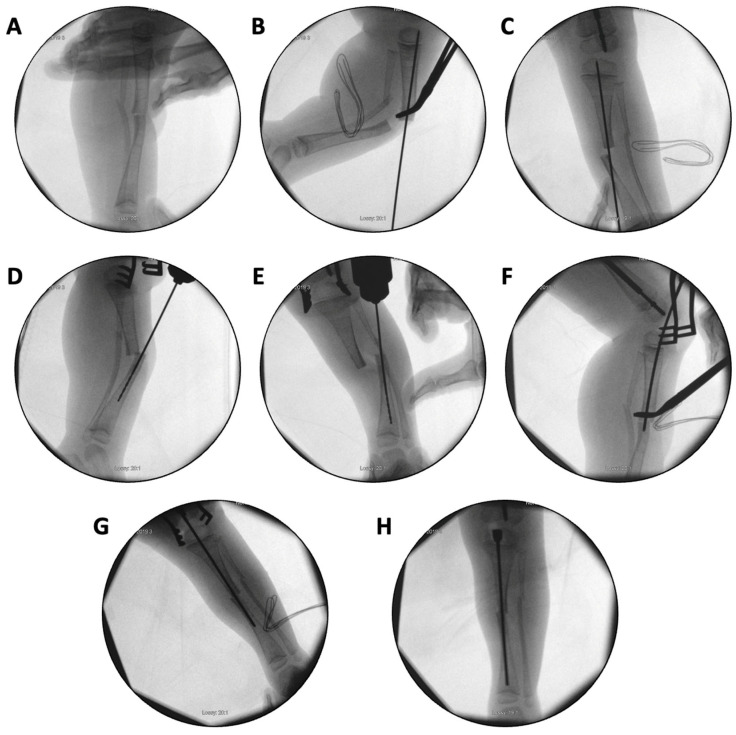
(**A**–**H**): Series of intraoperative fluoroscopy images demonstrating insertion of the SLIM nail. (**A**) After osteotomy, the fracture is reduced. (**B**) Lateral view of proximal reaming through the osteotomy site. (**C**) AP view of proximal reaming through the osteotomy site. (**D**) Lateral view of distal reaming through the osteotomy site. (**E**) AP view distal reaming through the osteotomy site. (**F**) Insertion of the SLIM nail. (**G**) SLIM nail is gradually advanced past the osteotomy site. (**H**) AP view of installed SLIM nail, with proximal threading in the epiphysis.

In the first case, a three-year-old skeletally immature girl diagnosed with OI type III presented for surgical correction of significant right tibial bowing using a SLIM nail. After identifying the CORA (Figure 8), an anterior opening-wedge osteotomy was performed at the midshaft of the right tibia to realign the bone (Figure 9A). The proximal canal was sequentially reamed from the osteotomy site to accommodate the SLIM nail, and a medial parapatellar approach was used to retrieve the reamer at the level of the knee (Figure 9B,C). The distal tibia was similarly reamed up to the distal physis (Figure 9D,E). The SLIM nail was inserted through the knee and positioned centrally within the canal with good bony contact on all sides, with the proximal threading positioned in the epiphysis (Figure 9F–H). Fluoroscopy confirmed excellent alignment, and no additional internal fixation was needed. In this case, the indication for using a SLIM nail was the narrow intramedullary canal of the tibia in a skeletally immature patient requiring deformity correction. A larger nail could not be used due to anatomical limitations, making the SLIM nail the appropriate choice.

**Figure 10:**
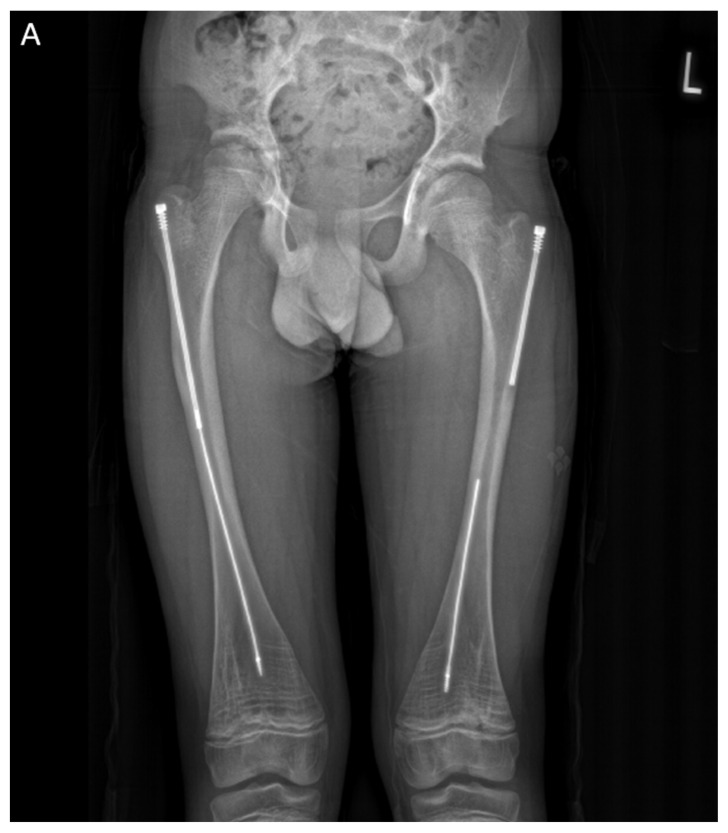

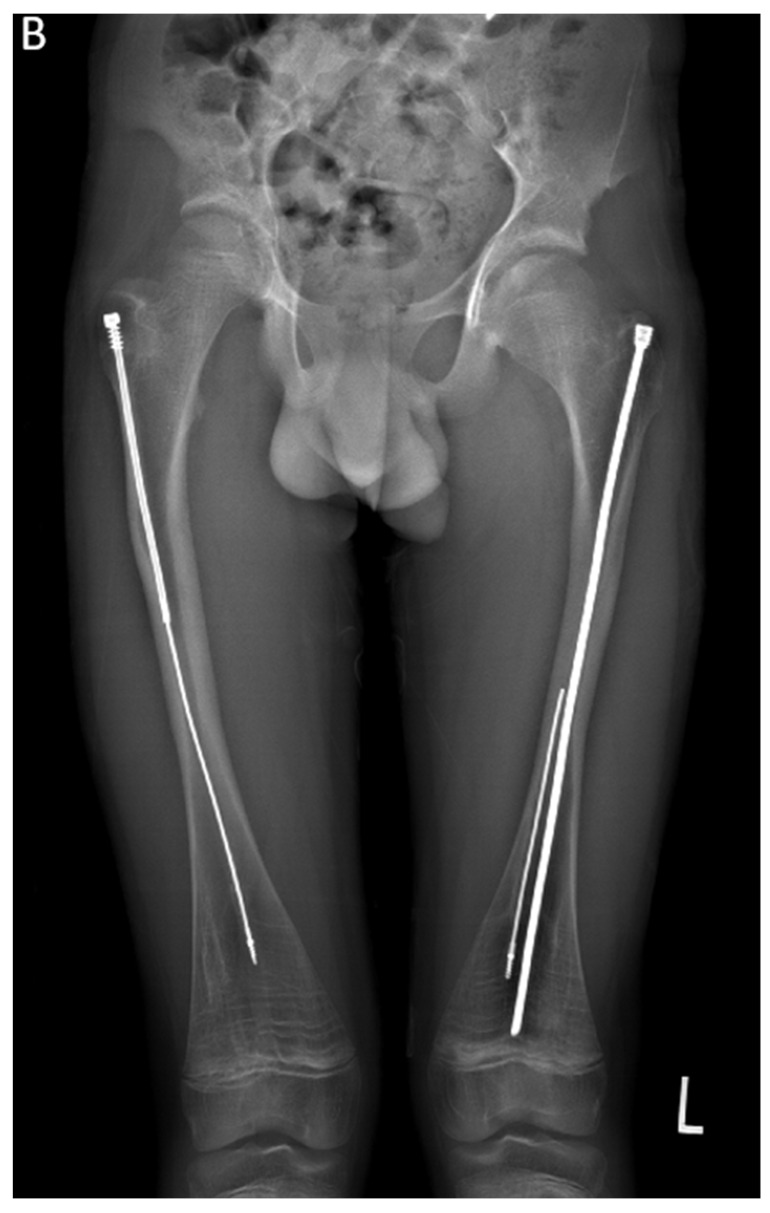
(**A**)—Pre-operative X-ray. (**B**)—Post-revision X-ray showing installed SLIM nail in the left femur.

In the second case presented in this paper, a 15-year-old boy diagnosed with OI type IV, who was previously operated on 12 years ago with bilateral FD rods, presented to his regular clinic visit with pain over the left greater trochanter. This patient was ambulating without any braces or aids and was functioning very well. He did not sustain any fractures since the FD rod insertion, and full ROM of his hips was maintained. X-ray showed a completely disengaged FD rod on the left side, where the female component migrated distally and laterally across the lateral cortex. The protruding proximal part of the female component was the site of the pain and tenderness (Figure 10A). On the right side, the FD rod was almost disengaged, and a mild deformity was present anterolaterally. The X-ray demonstrates that the intramedullary canal is too narrow to accommodate an adult-sized nail. As the patient had almost reached skeletal maturity, it was decided not to use another telescoping rod but instead to use a non-telescoping SLIM nail. Because the distal male component of the existing FD rod was situated deep into the canal, making its retrieval problematic, it was decided pre-operatively to leave it in situ and insert the new rod beside it. The female component on the left side needed to be removed, and a solid SLIM rod was inserted to protect the bone (Figure 10B). Revision to a non-telescoping rod was considered for the right side as well; however, surveillance was opted for as the patient was approaching skeletal maturity. In this case, the indication for use of the SLIM nail is a near skeletally mature patient, therefore, not requiring a telescoping nail. However, the intramedullary canal was too narrow to accommodate a larger solid nail.

**Figure 11:**
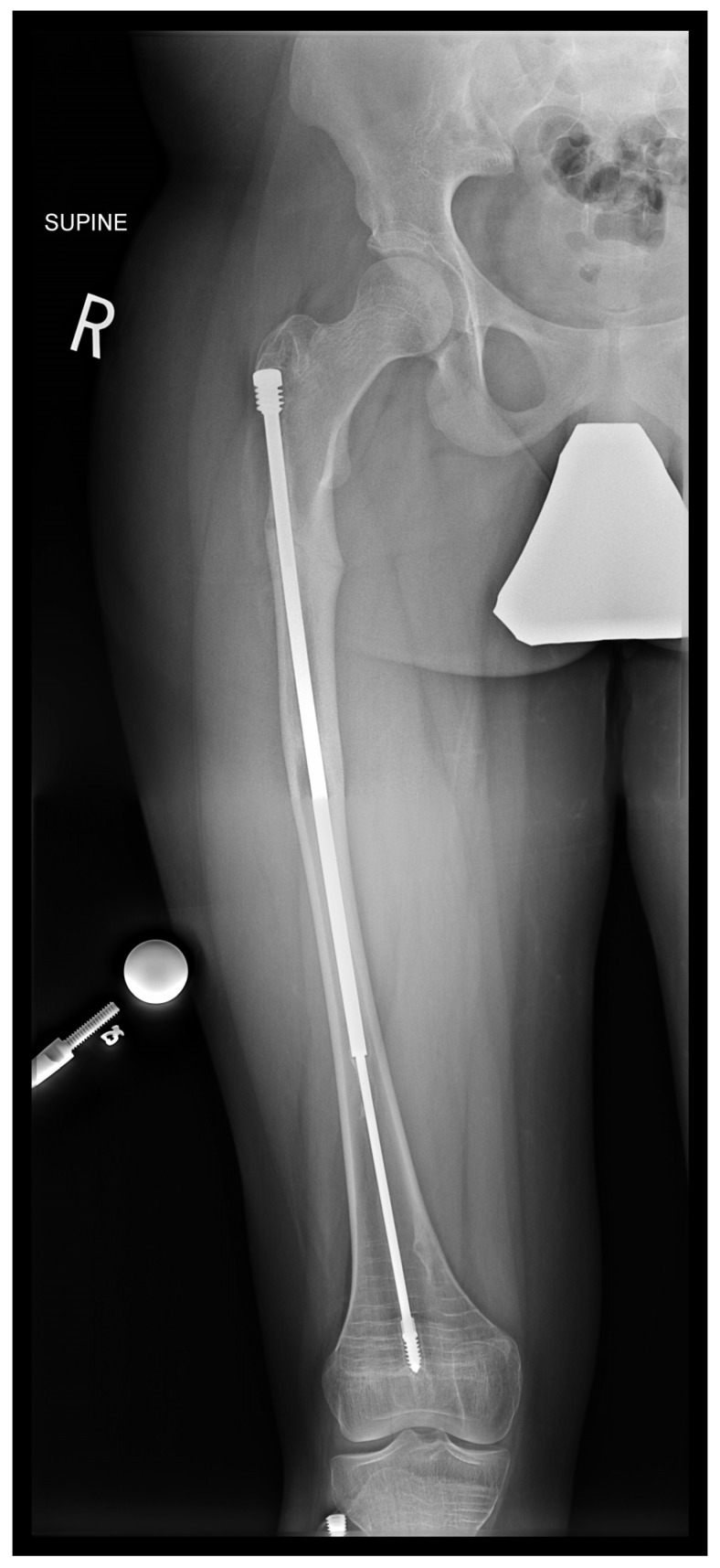
Pre-operative X-ray showing right femur stress fracture.

**Figure 12:**
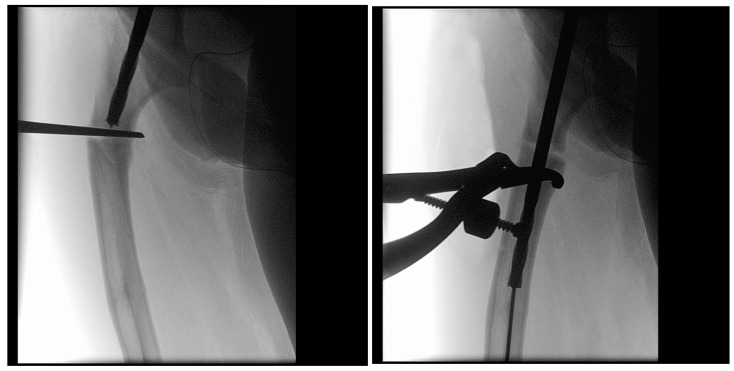
Intraoperative X-rays showing the first CORA and alignment with the continuation of the reaming.

**Figure 13:**
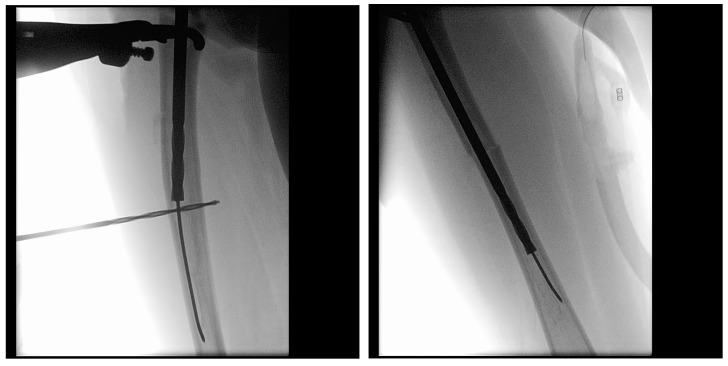
Intraoperative X-rays showing the second CORA and realignment with the continuation of the reaming.

**Figure 14:**
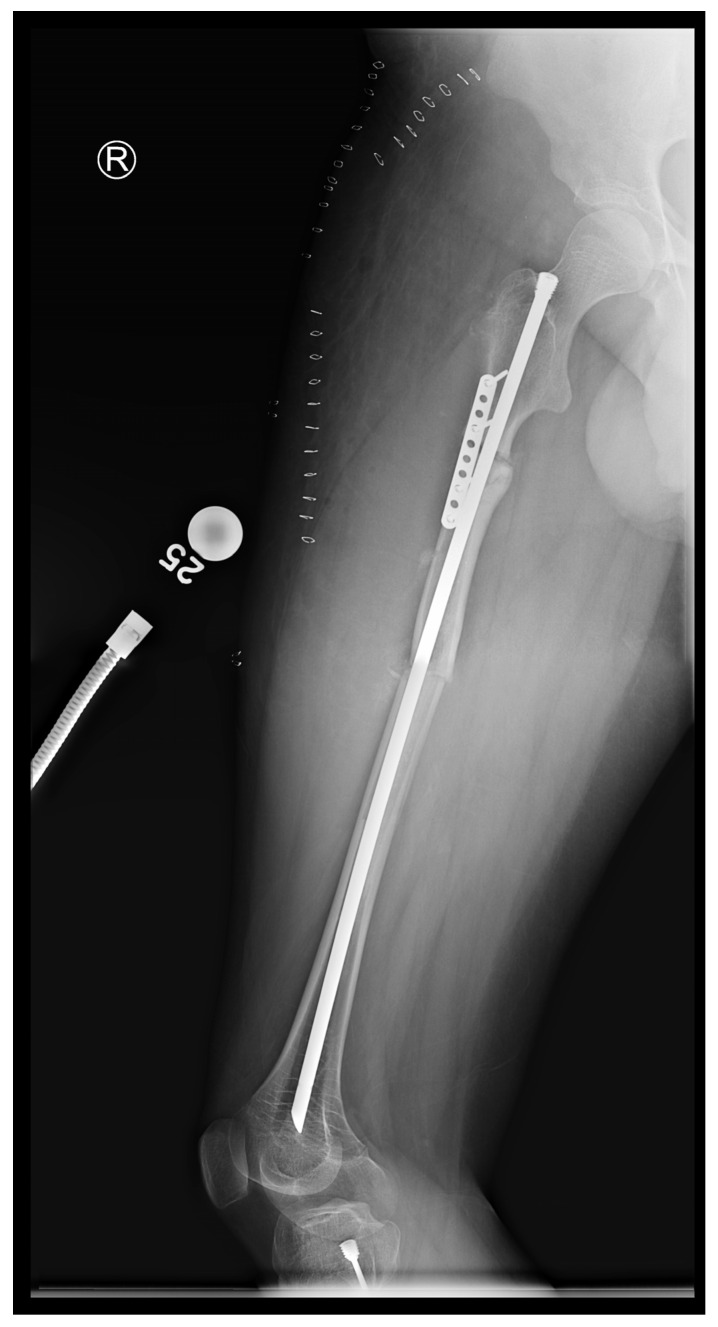
Post-operative X-ray showing SLIM nail and plate.

In the third case, a 16-year-old skeletally mature patient with OI was scheduled to undergo revision from FD rod to SLIM nail after presenting with pain due to stress fractures and wild deformity in his proximal right femur (Figure 11). The FD rod was removed, and then, using a piriformis entry point, access to the femur and proximal reaming was performed. There were two CORAs; the first CORA was identified in the subtrochanteric region, and a percutaneous osteotomy technique was performed to realign the bone, and then reaming was continued (Figure 12). The second CORA was identified, and another percutaneous osteotomy was performed, and the bone was cracked with a closed osteoclasis technique, and then reaming was continued in the distal segment (Figure 13). The SLIM nail was then inserted and had an excellent fit. The subtrochanteric region was under high stress, so a 2.7 Smith and Nephew EVOS plate to further stabilize the segment and control the rotation. Two screws were placed above and below (Figure 14). In this case, the indication for the use of the SLIM nail was the need to revise the existing telescoping FD rod in a skeletally mature patient to a solid nail. As the intramedullary canal was too small to accommodate a larger solid nail, a SLIM nail was used instead. The use of a plate as an adjunct to an intramedullary nail is also shown.

These three cases were selected as they represent the most common indications and surgical pearls for use of the SLIM nail: (1) skeletally immature patients with narrow IM canals, (2) revision of telescoping rod to solid rod in skeletally mature patients with narrow IM canals, and (3) the adjunct use of plating. (Table 1).

## 4. Discussion

The success of SLIM nail implantation relies not just on selecting the appropriate implant but on thoughtful surgical planning and precise intraoperative execution. Pediatric patients with osteogenesis imperfecta (OI) pose unique anatomical and mechanical challenges: their bones are fragile, osteopenic, narrowed, often sclerotic, and frequently deformed, with small intramedullary canals, multiple centers of rotation of angulation (CORAs), and thin cortices [8,9]. These features increase the risk of iatrogenic fracture, eccentric reaming, and hardware failure, requiring the surgeon to carefully adapt technique to each patient’s anatomy.

The SLIM nail is primarily indicated in two main scenarios: first, as an initial treatment for patients whose narrow canals are unsuitable for telescoping rods like the FD system, and second, as a revision implant for skeletally mature patients who no longer require elongating implants but whose canal is too narrow to accommodate larger solid nails In the three cases presented, we illustrate common applications of the SLIM nail: Case 1 demonstrated successful tibial bowing deformity correction in a skeletally immature patient who’s IM canals were too small to accommodate a telescoping rod, Case 2 involved revision to a solid nail in a near-mature patient whose narrow canal prohibited the use of larger adult nails, and Case 3 required correction at two CORAs with adjunctive plate fixation to manage excessive rotational instability.

Several technical pearls proved to be important across these cases. Optimizing patient positioning, such as internally rotating the limb to achieve a clear lateral view without constant C-arm adjustments, improved intraoperative efficiency. Precise, central guidewire placement was essential in narrow or sclerotic canals to prevent eccentric reaming and cortical perforation. To avoid thermal damage, multiple drill holes combined with sharp osteotomes were used, rather than oscillating saws, helping preserve bone healing potential. In cases where intramedullary fixation alone was insufficient, supplemental plating was used to add necessary stability. These pearls are not minor technical details but essential strategies that help minimize intraoperative risk and ultimately improve postoperative outcomes. By sharing these practical insights, we aim to assist other surgeons navigating the complex anatomical and surgical challenges posed by OI patients undergoing SLIM nailing.

While the SLIM nail addresses several limitations of older non-telescoping rods like K-wires, Rush rods, and Enders nails, it remains prone to certain complications. In a prior institutional cohort study of 23 patients (41 limbs), we observed an implant survival rate of 82.8% at 2 years and 77.1% at 4 years. These rates compare favorably to previous studies using static rods, where survivorship ranged from 36% to 52% at similar time points [10,11]; however, they are lower than survivorship rates reported for telescoping nails, which range from 77% to 92.9% [12,13]. Therefore, in borderline canals that can safely accept the smallest FD rod, a telescoping rod is still preferred. When reaming would compromise bone stock, the SLIM nail provides stability while its threaded head engages the proximal epiphysis of the bone and prevents it from migration. The most common complication with the SLIM nail is angular deformity in the distal unprotected portion of growing bones, particularly the tibia [14]. Other complications include anterior cortical penetration, nail bending, breakage, and non-union [14]. Therefore, regular radiographic surveillance is essential to plan timely revisions and avoid preventable complications. Notably, revision indications were not limited to implant outgrowth but also included fractures (displaced or repeated), angular deformity over 25 degrees, pain, or hardware failure. Our experience aligns with the broader literature on non-telescoping intramedullary rods. Scollan et al.’s meta-analysis of 359 non-elongating intramedullary rod procedures reported a rod migration rate of 25.7% and a fracture rate of 15% following static rod placement [15]. Imajima et al. evaluated 29 femoral K-wire implants and recommended revision when the wire-to-bone length ratio approached 70%, noting a sharp increase in wire-tip fractures once the ratio dropped below this threshold [10].

The presented examples emphasize how adapting surgical technique to the specific anatomical and mechanical challenges of OI patients is critical for success with SLIM nail implantation. Continued experience and future research will be essential to refine indications, reduce complications, and improve long-term outcomes for this demanding patient population.

## 5. Conclusions

The SLIM nail broadens the surgical options available for managing osteogenesis imperfecta, especially in patients with narrow medullary canals or those requiring revision as they near skeletal maturity. However, its successful use depends not only on implant selection but on meticulous surgical technique, including precise guidewire placement, careful osteotomy planning, and the addition of supplemental fixation when needed. The technical pearls outlined in this manuscript are intended to help minimize complications, improve implant stability, and enhance postoperative outcomes. By sharing these practical insights, we aim to provide useful guidance for surgeons working in this demanding surgical context.

## Figures and Tables

**Table 1:** Summary of the three SLIM Nail cases.

Case	Age	Type of OI	Bone (s)	Plate	Indication/Surgical Pearls
1	3 years	III	Tibia	N	SLIM nail insertion in a skeletally immature patient with a narrow IM canal to correct significant bowing; reaming performed retrograde from the osteotomy site for the proximal segment and antegrade for the distal segment.
2	15 years	IV	Femur	N	Revision to SLIM nail in a near-mature OI patient with FD rod disengagement and narrow IM canal; retained distal hardware and inserted SLIM rod alongside.
3	16 years	V	Femur	Y	Revision of FD rod to SLIM nail in a skeletally mature patient with narrow IM canal; dual percutaneous osteotomies at two CORAs and adjunct plating for alignment and stability.

## Data Availability

The original contributions presented in this study are included in the article.Further inquiries can be directed to the corresponding author(s).
